# Inactivated Eyedrop Influenza Vaccine Adjuvanted with Poly(I:C) Is Safe and Effective for Inducing Protective Systemic and Mucosal Immunity

**DOI:** 10.1371/journal.pone.0137608

**Published:** 2015-09-10

**Authors:** Eun-Do Kim, Soo Jung Han, Young-Ho Byun, Sang Chul Yoon, Kyoung Sub Choi, Baik Lin Seong, Kyoung Yul Seo

**Affiliations:** 1 Department of Ophthalmology, Eye and Ear Hospital, Severance Hospital, Institute of Vision Research, Yonsei University College of Medicine, Seoul, Republic of Korea; 2 Brain Korea 21 Project for Medical Science, Yonsei University, Seoul, Republic of Korea; 3 Department of Biotechnology, College of Engineering, Yonsei University, Seoul, Republic of Korea; 4 The Graduate School of Yonsei University, Seoul, Republic of Korea; 5 Vaccine Translational Research Center, Yonsei University, Seoul, Republic of Korea; 6 Department of Ophthalmology, National Health Insurance Corporation Ilsan Hospital, Gyounggi-do, Republic of Korea; The University of Adelaide, AUSTRALIA

## Abstract

The eye route has been evaluated as an efficient vaccine delivery routes. However, in order to induce sufficient antibody production with inactivated vaccine, testing of the safety and efficacy of the use of inactivated antigen plus adjuvant is needed. Here, we assessed various types of adjuvants in eyedrop as an anti-influenza serum and mucosal Ab production-enhancer in BALB/c mice. Among the adjuvants, poly (I:C) showed as much enhancement in antigen-specific serum IgG and mucosal IgA antibody production as cholera toxin (CT) after vaccinations with trivalent hemagglutinin-subunits or split H1N1 vaccine antigen in mice. Vaccination with split H1N1 eyedrop vaccine antigen plus poly(I:C) showed a similar or slightly lower efficacy in inducing antibody production than intranasal vaccination; the eyedrop vaccine-induced immunity was enough to protect mice from lethal homologous influenza A/California/04/09 (H1N1) virus challenge. Additionally, ocular inoculation with poly(I:C) plus vaccine antigen generated no signs of inflammation within 24 hours: no increases in the mRNA expression levels of inflammatory cytokines nor in the infiltration of mononuclear cells to administration sites. In contrast, CT administration induced increased expression of IL-6 cytokine mRNA and mononuclear cell infiltration in the conjunctiva within 24 hours of vaccination. Moreover, inoculated visualizing materials by eyedrop did not contaminate the surface of the olfactory bulb in mice; meanwhile, intranasally administered materials defiled the surface of the brain. On the basis of these findings, we propose that the use of eyedrop inactivated influenza vaccine plus poly(I:C) is a safe and effective mucosal vaccine strategy for inducing protective anti-influenza immunity.

## Introduction

For immunization against influenza, there are two major routes of vaccination: muscular injection and intranasal (IN) administration. Parenteral injection is the most widely and traditionally used method in almost all vaccine regimens; nevertheless, such injections mainly induce serum IgG antibody without inducting secretion of IgA to mucosal surfaces of the respiratory tract, which is the main infection route of the influenza virus. In contrast, intranasal administration induces both systemic IgG and mucosal secretory-IgA (S-IgA) production, initiating mucosal immunity; therefore, intranasal vaccination is more potent than parenteral injection for the prevention of influenza [1, 2]. Moreover, IN vaccination is advantageous in that is does not require the use of syringes, enabling anyone to readily administer the vaccine without special training.

Recently, some nasal spray live-attenuated influenza vaccines (LAIV), such as FluMist, were approved by the Food and Drug Administration (FDA) for human use in the United States. However, LAIV can cause some side effects such as sore throat, coryza, and febrile reactions [3]. As a result, it is not allowed for use in pregnant woman and immunodeficient patients, as well as in children under the age of 12 months [4] or adults over 50 [5]. Therefore, two major high-risk groups are excluded from vaccination with the live-virus vaccine. Meanwhile, studies showed that if the inactivated influenza vaccines are intranasally administered, it can induce nerve damage with olfactory bulb (OB)-mediated antigen and adjuvant diffusion into the brain, in the presence of cholera toxin (CT) adjuvant [6, 7]. Moreover, introduction of inactivated intranasal influenza vaccine reportedly provoked Bell’s palsy in human [8]. Thus, many studies have attempted to devise alternative ways of inducing mucosal immunity to circumvent the side effects of the intranasal influenza vaccines.

Lately, the eye mucosa has come to the forefront as a promising vaccination route. The eye mucosa, which exhibits the common immunological structures of mucosal tissues, including conjunctiva-associated lymphoid tissue (CALT) [6, 9–11] and tear-associated lymphoid tissue (TALT) [12, 13], is an inductive site for the acquirement of systemic and mucosal immunity. The early trials of eyedrop vaccination in avian and bovine models showed that eyedrop vaccination induces protective immunity against Newcastle disease virus and *Brucella melitensis*, respectively [14, 15]. Additionally, our group was the first to report the feasibility of the use of eyedrop influenza and *Salmonella* vaccines in mice [6]. Furthermore, eyedrop vaccination does not redirect antigen with cholera toxin (CT) into the CNS as in intranasal vaccination [6, 7].

Polyriboinosinic:polyribocytidylic acid (poly[I:C]), a ligand of mammalian toll-like receptor 3, a known receptor for double-stranded RNA, induces interferon alpha/beta production via activation of NF-κB pathways [16]. Poly(I:C), therefore, exerts adjuvant effects by linking the gap between innate and adaptive immunity by enhancing primary antibody responses [17]. Additionally, the mucosal adjuvant effect of poly(I:C) against influenza virus has also been shown in intranasally vaccinated inactive-influenza virus hemagglutinin (HA) vaccine plus poly(I:C) in mice [18]. Recently, the activity of poly-L-lysine stabilized poly(I:C) analogue (poly ICLC) as a vaccine adjuvant and innate-immunity activator was assessed in non-human primates and human models, respectively [19, 20]. Thus, clinical trials of the use of poly(I:C) and its safe analogue may demonstrate the possibility of using potent adjuvant in influenza vaccine immunization.

In our previous study, we reported that eyedrop vaccination with live-attenuated vaccines, such as influenza virus or *Salmonella* bacteria, can protect mice from lethal challenge of pathogens [6]. However, the vaccines used in the study were live-attenuated, which may cause unexpected side-effects. Therefore, examining the efficacy of inactivated vaccines immunized by the eye-route is necessary in order to circumvent the use of live-vaccine. However, since it is hard to induce a sufficient immune response by inactivated influenza vaccine-antigen alone, use of adequate adjuvants to induce complete protective immunity is critical to vaccination with inactivated-vaccine antigens.

Here, we show that poly(I:C) is a potent adjuvant, except for CT, for use in eye-route vaccination for the immunization of killed-influenza virus vaccine antigen. Administration of inactivated influenza vaccine plus poly(I:C) by eyedrops exerted significantly enhanced production of Ag-specific Ab in both systemic and mucosal immunity, by which mice were protected from lethal influenza virus challenge. Also, there was no signs of inflammation in the eyes after poly(I:C) was administered. Moreover, administration of vaccine materials by eyedrops did not contaminate the surface of the brain in contrast to IN, which defiled olfactory bulb regions of the mouse brain. On the basis of our findings, we propose that eyedrop vaccination of killed-influenza vaccine along with poly(I:C) is an effective and safe preventive measure to induce protective immunity against influenza virus infection.

## Materials and Methods

### Mice

This study was performed in strict accordance with the recommendations in the Guide for the Care and Use Committee of Yonsei University Health System. The committee has reviewed and approved the animal study protocol (Approval No: 2011–0137). Specific pathogen-free female BALB/c mice, aged 6–10 weeks, were purchased from Charles River Laboratories (Orient Bio, Sungnam, Korea). All mice were maintained in the experimental animal facility under specific pathogen-free conditions at Yonsei College of Medicine (Seoul, Korea) and received sterilized food (Certified Diet MF; Oriental Yeast, Osaka, Japan) and filtered tap water ad libitum. All surgeries were performed after sacrificed by CO2 narcosis and every effort was made to minimize suffering.

### Vaccine Antigens and Adjuvants

The influenza virus subunit vaccines were provided by Dr. Na Gyong Lee (Sejong University, Seoul, Korea). The trivalent HA vaccine comprised the HA subunits from three influenza virus strains: A/New Caledonia/20/99 (H1N1), A/Panama/2007/99 (H3N2) and B/Shandong/7/97. The split H1N1 influenza virus vaccine was provided by Green Cross Co. (Yongin, Gyeonggi, Korea). The vaccine comprised the split influenza A/California/7/2009 (NYMCX-181) (H1N1) virus. Various adjuvants were used including cholera toxin (CT; List Biological Laboratories, Campbell, CA), polyinosinic-polycytidylic acid (poly(I:C), monophosphoryl lipid A (MPLA) from *Salmonella minnesota*, CpG oligonucleotide (InvivoGen, San Diego, CA), and ImjectAlum (Thermo Scientific, Rockford, IL).

### Immunization

Prior to experimental manipulation, 6–10-week-old female mice were anesthetized by i.p. injection of zoletil (30 mg/kg body weight) and rompun (10 mg/kg body weight). For conjunctival immunization, 100 μg of ovalbumin (OVA) (Sigma-Aldrich, St. Louis, MO) and various adjuvants (0.1 μg to 10 μg poly(I:C) or 10 μg MPLA or 2 μg CT or 1 mg Imject Alum) were suspended in 5 μl of phosphate-buffered saline (PBS) and dropped weekly for 3 consecutive weeks onto both conjunctival sacs by a micropipette. In some experiments, mice were immunized with 1 μg of trivalent-HA vaccine or 1 μg of H1N1 split vaccine with various adjuvants resuspended in 15 μl or 10 μl of PBS, respectively, and subsequently dropped once more at a 2-week interval. For HA antigen-dose dependent immunization experiments, 0.5 μg or 1 μg of HA antigens plus 10 μg poly(I:C) were resuspended in 10 μl of PBS, and 2 μg of HA antigens plus 10 μg poly(I:C) were resuspended in 15 μl of PBS. For IN immunization, mice were administered with 1 μg of trivalent-HA vaccine or 1 μg of H1N1 split vaccine with various adjuvants resuspended in 20μl of PBS and subsequently vaccinated once more at a 2-week interval. For long-term immunity induction experiments, mice were immunized with 1 μg of H1N1 split vaccine plus 10 μg of poly(I:C) resuspended in 10 μl of PBS three times at 2-week intervals.

### Sample Collection

At two weeks or one year after the final immunization, serum was obtained by retro-orbital bleeding. Tear-wash samples were obtained by lavaging with 10 μl of PBS per eye. Saliva was obtained following i.p. injection of mice with pilocarpine (500 mg/kg body weight; Sigma-Aldrich). Fecal extract was obtained by adding weighed feces to PBS containing 0.1% sodium azide. The feces samples were mixed by vortexing and subsequently centrifuged, and the supernatants were collected for assay. Vaginal wash samples were collected by lavage with 100 μl of PBS. To obtain bronchoalveolar lavage fluid (BALF), tracheas were cannulated after exsanguination, and the lungs were washed with 1 ml of PBS. After the mice were sacrificed, nasal wash samples were obtained by flushing 100 μl of PBS through the anterior (oral) entrance of the nasal passages using a pipette.

### cDNA Synthesis and Real-Time Quantitative PCR

After a wash with nuclease-free water, we homogenized the whole conjunctival or corneal tissue samples at different time points after eye-drop immunization with HA vaccine alone (1 μg), HA plus poly(I:C) (10 μg), or HA plus CT (2 μg). Total RNA was extracted using TRIzol (Invitrogen), and cDNA was synthesized by Superscript II reverse transcriptase with oligo (dT) primer (Invitrogen). cDNA was amplified with HotStart-IT SYBR Green qPCR Master Mix (USB, Cleveland, OH) and gene-specific forward and reverse primers on an ABI 7300 Real- Time PCR system (Applied Biosystems). Results are expressed as mean ± S.D. after normalizing to the expression of β-actin gene using the ΔΔCt method. Primer sequences are available upon request.

### ELISA for Detection of Ag-Specific Ab

ELISA plates (Nunc, Roskilde, Denmark) were coated with OVA (100 μg/ml) or HA vaccine antigen (2 μg/ml) or H1N1 split vaccine (1 μg/ml) in coating buffer and incubated overnight at 4°C. Blocking was done with 1% bovine serum albumin (Sigma-Aldrich) in PBS, and two-fold serially diluted samples were applied to plates. HRP-conjugated goat anti-mouse IgG or IgA Ab (Southern Biotechnology Associates, Birmingham, AL) was added to each well and incubated overnight at 4°C. For color development, tetra-methylbenzidine solution (Thermo Scientific, Rockford, IL) was used. Plates were then measured at 450 nm on an ELISA reader (Molecular Devices, Sunnyvale, CA) after addition of stopping solution (0.5 N HCl). Endpoint titers of Ag-specific Ab were expressed as reciprocal log_2_ titers of the last dilution that showed > 0.1 absorbance over background levels.

### Protection Assay against Influenza Virus A/California/04/09

At two weeks or one year after the final eyedrop or intranasal immunization with 1 μg of H1N1 split vaccine alone or plus 10 μg of poly(I:C), five mice in each group were anesthetized and challenged with 50 μl of mouse-adapted live influenza A/California/04/09 (H1N1) virus suspension (10 X LD_50_; 0.75 TCID_50_) via the IN route. Animals were monitored for weight loss and survival every day for 14 days, and there were 3 unexpected deaths in PBS or H1N1 antigen alone administered groups. The specific clinical signs we used to determine when the animals should be euthanized was the loss of 20% of initial bodyweights. Euthanasia was done by CO2 inhalation with a fill rate of about 10% to 30% of the chamber volume per minute with carbon dioxide, added to the existing air in the chamber.

### Plaque Assay

The measurement of the viral titers in lung tissues in virus-challenged mice were measured as previously described [21]. Briefly, the viral titers used in this study were expressed as the plaque forming units (PFU) calculated by plaque assay. Monolayers of Madin-Darby Canine Kidney cells in 12-well-plates were infected with 10-fold serial dilutions of virus solutions for 45 min at room temperature. After the removal of the solutions, the cells were washed with PBS and overlaid with minimum essential medium, containing 1% low melting agarose (Lonza, ME, USA) and 10 μg/ml of trypsin (Life Technology, NY, USA). After the overlay turned solid, the plates were moved to an incubator with 5% CO2. The plaques formed were fixed by formaldehyde solution and visualized by staining with crystal violet.

### Histology

Eye tissues including the conjunctiva and eye balls from controls and 1 μg HA vaccine plus 10 μg poly(I:C) or 2 μg CT treated mice were washed with PBS and fixed in 4% formaldehyde for 24 h at 4°C. The tissues were dehydrated by gradual soaking in alcohol and xylene and then embedded in paraffin. The paraffin-embedded specimens were cut into 5-mm sections and stained with H&E.

### Micro-CT

Imaging was performed as previously described [22] with minor modification using a volumetric CT scanner (NFR-Polaris-G90MVC: NanoFocusRay, Iksan, Korea). Briefly, images were acquired at 65 kVp, 115 μA, and 142-millisecond per frame, and for 700 views. The estimated radiation dose was ~150 mGy using this image acquisition protocol. Images were reconstructed using the volumetric cone-beam reconstruction (FDK) off line mode. The size of reconstruction images was 1,204 x 1,024 pixels, and 512 slices were acquired. The final reconstructed data were converted to the Digital Imaging and Communications in Medicine (DICOM) format to generate 3D-rendered images using 3D-rendering software (Lucion, MeviSYS, Seoul, Korea). For *ex vivo* brain CT imaging, all BALB/c mice were sacrificed, and the brains of each ocularly or intranasally treated mouse were taken out and CT images thereof were acquired.

### Data and Statistical Analyses

Data were expressed as the mean ± SD, and statistical analyses were conducted by the ANOVA test (Microsoft Office Excel program).

### Ethics Statement

All experiments involving animal subjects were conducted in strict accordance and adherence to relevant national and international guidelines regarding animal handling as mandated by the Institutional Animal Care and Use Committee (IACUC) of Yonsei University Health System (Seoul, Korea). Approval number is 2011–0137.

## Results

### Significant Enhancement of Ag-Specific Ab Production by Eyedrop Influenza Vaccine Adjuvanted by Poly(I:C)

**Fig 1:**
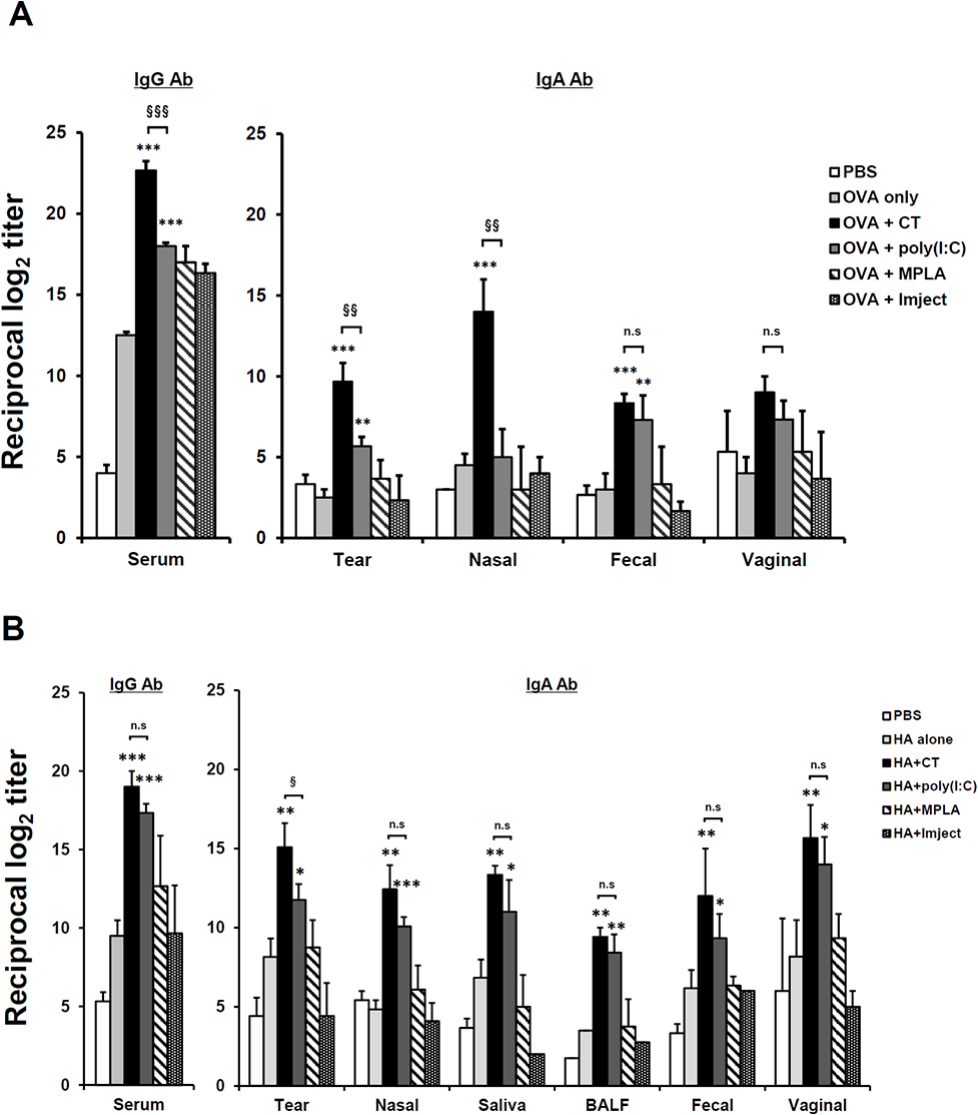
CT and Poly(I:C) enhances systemic and mucosal Ab production by eyedrop vaccination of OVA or HA. Groups of female BALB/c mice received OVA (100 μg) (A) or HA (1 μg) (B) plus CT (2 μg), poly(I:C) (10 μg), MPLA (10 μg), Imject Alum (320 μg) resolved in 5 μl of PBS, or 5 μl of PBS alone by drops on both eyes every week (three times). Ag-specific Ab levels were measured in serum and in various mucosal fluids 1 wk after final vaccination by ELISA. * *p* < 0.05, ** *p* < 0.01, *** *p* < 0.001 versus Ag alone; §§ *p* < 0.01, §§§ *p* < 0.001 versus poly(I:C) group; ‘n.s.’, non-significant. Results are representative of three independent experiments, with three mice in each group.

To evaluate the efficacy of various adjuvants in regards to whether they can enhance systemic and mucosal antibody production when used with protein antigen by eye-route vaccination, BALB/c mice were immunized with OVA (100 μg/head) protein plus several conventionally used adjuvants, including CT (2 μg/head), poly(I:C) (10 μg/head), MPLA (10 μg/head), or Imject Alum (1 mg/head), which is a commercially used alum adjuvant, three times at one-week intervals. One week after final immunization, the levels of OVA-specific Abs were measured by ELISA. All mice given OVA plus adjuvant showed significantly higher levels of OVA-specific serum IgG Ab in there sera than those found in mice given PBS or OVA alone (Fig 1A). Among the adjuvants, CT showed the highest serum IgG Ab production level. However, only mice given OVA plus CT or poly(I:C) showed significantly higher levels of IgA Ab in mucosal compartments (e.g., tear, nasal, fecal, and vaginal washes) than the other adjuvant-treated groups.

To examine whether the Ab production-enhancing effect adjuvanted by CT or poly(I:C) was retained in influenza subunit-vaccine immunized mice, as shown in Fig 1A, BALB/c mice were immunized with 1 μg of the seasonal influenza vaccine, consisting of trivalent HAs of A/New Caledonia/20/99 (H1N1), A/Panama/2007/97 (H3N2), and B/Shandong/7/97 influenza (B) viruses, with the same adjuvants that were previously used with OVA protein. As shown in Fig 1B, Ab production levels for serum IgG and mucosal IgA Ab in mice treated with CT or poly(I:C) were significantly higher than those for the other groups. The enhancing effects of MPLA and the Imject adjuvants were not observed for either types of Abs, compared to mice given PBS or Ag alone. Unexpectedly, the efficacy of Abs-production enhancement of poly(I:C) was similar to that of CT. Moreover, poly(I:C) significantly improved the production of mucosal IgA Ab to as much as CT did in the respiratory passageways, in nasal cavity wash and BALF samples, which is the main route of influenza infection, indicating that it could be of use as a front-line defense against influenza invasion [23]. While CT has been shown to cause CNS damage when it was administrated intranasally [7, 8], there is a report of a lack of a destructive effect on nasal or brain tissue after administration of poly(I:C) [18]. Therefore, in the following experiments, we focused on assessing the adjuvanticity of poly(I:C) via eyedrop administration.

### Dose-Dependent Ab Responses in Eyedrop Vaccines with Various Amounts of Ag and Poly(I:C)

**Fig 2:**
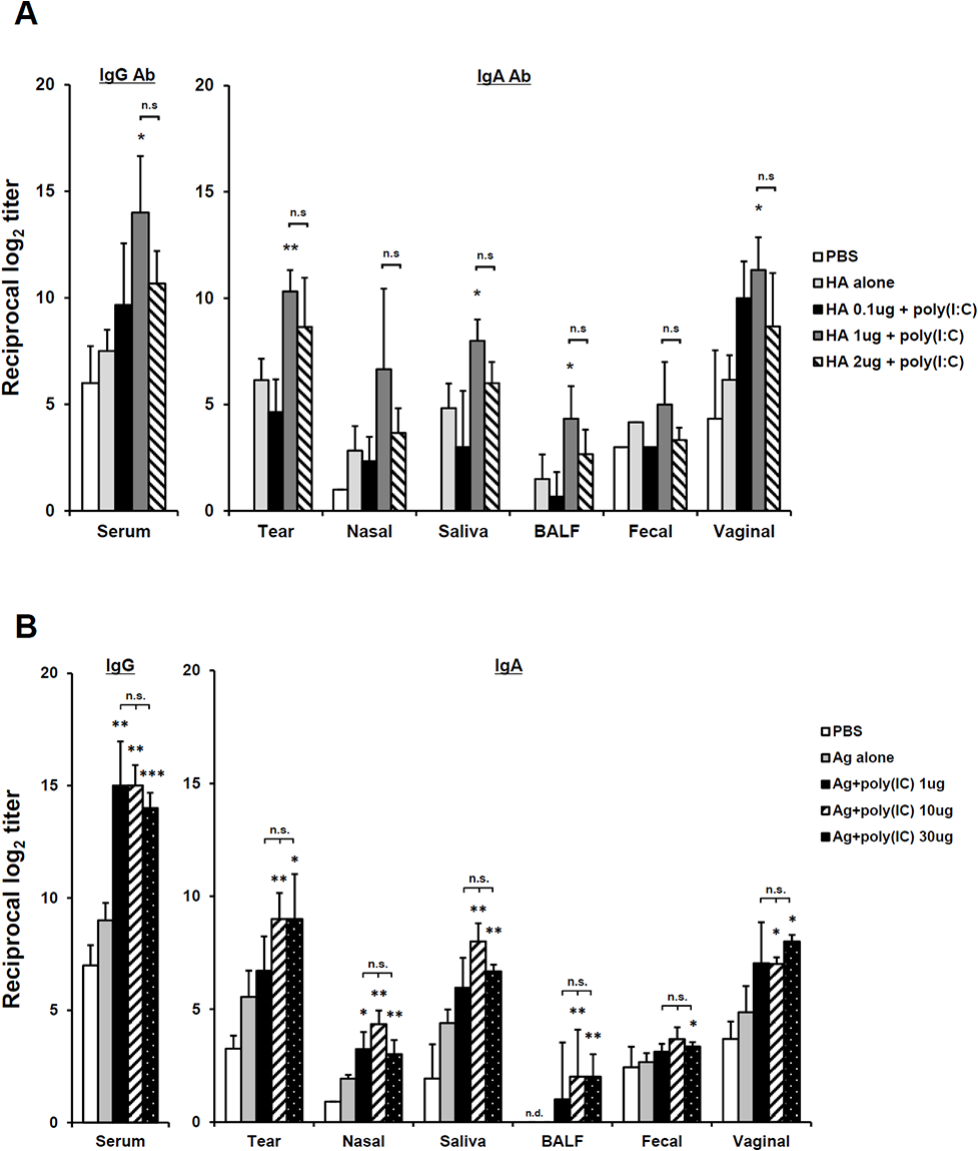
Comparison of antibody production by the amount of hemagglutinin (HA) or poly(I:C). (A). Dose-dependent HA vaccine antigens were administered with 10 μg poly(I:C) resolved in 5 μl of PBS by drops on both eyes two times at a 2-week interval in female BALB/c mice. HA-specific Ab levels were measured in serum and in various mucosal fluids 2 weeks after final vaccination by ELISA. (B) 1 μg of HA vaccine antigen plus dose-dependent poly(I:C) was vaccinated by drops on both eyes two times at a 2-week interval in female BALB/c mice. HA-specific Ab levels were measured in serum and in various mucosal secretions 2 weeks after final vaccination by ELISA. * *p* < 0.05; ** *p* < 0.01 versus Ag alone group; ‘n.s.’, non-significant. Results are representative of three independent experiments, with three mice in each group.

Next, we attempted to elucidate the minimum amount of Ag and poly(I:C) that could effectively induce the enhancement of systemic and mucosal Ab production via eyedrop vaccination. To do so, BALB/c mice were immunized with 10 μg of poly(I:C) and various amounts of HA vaccine, 0.1 μg, 1 μg or 2 μg, via eyedrop vaccination (Fig 2A). Since the maximum volume of eyedrops per eye is 15 μl and the concentration stocks of HA vaccine and poly(I:C) were 1 μg per 11 μl and 10 μg per 4 μl, respectively, we could not examine the effect of antigen amounts over 2 μg HA vaccine plus 10 μg poly(I:C) in the eyedrop vaccination. As shown in Fig 2A, 1 μg of HA antigen plus 10 μg poly(I:C) significantly enhanced serum IgG and mucosal IgA Ab production amounts greater than those for mice given doses of 0.1 μg or 2 μg antigen plus 10 μg poly(I:C). Unexpectedly, the enhancement from 2 μg HA eyedrops with 10 μg poly(I:C) was less effective than that from 1 μg HA vaccine antigen eyedrops. Since the total volume of 1 μg or 2 μg HA antigens plus 10 μg poly(I:C) were 10 μl or 15 μl, respectively, it was estimated that the concentration of poly(I:C) was diluted in the doubled volume of 2 μg HA Ag eyedrops and, accordingly, the effect of poly(I:C) was diminished, compared to that for 1μg Ag plus 10 μg poly(I:C) eyedrops. Thus, for followed experiments, we used 1 μg of HA vaccine antigen for eyedrop vaccination.

After discovery of the effective minimum amount of HA vaccine antigen in the eyedrop vaccination, we checked the dose-dependent efficacy of poly(I:C) in the 1 μg HA eyedrop vaccine in mice. The levels of HA-specific Ab production in all groups of mice in which poly(I:C) was administered together with 1 μg HA-Ag eyedrop vaccine were significantly enhanced, compared to mice treated with 1 μg HA-Ag alone in both serum and the mucosal washes (Fig 2B). Additionally, there was almost no difference in the adjuvant efficacies of all poly(I:C) administered groups; even administration of 1 μg of poly(I:C) significantly augmented Abs production levels in the 1 μg of HA-Ag eyedrop vaccinated mice to as much as those for mice treated with 30 μg of poly(I:C). Thus, these results indicate that the efficacy of 1 μg poly(I:C) was enough to induce significant Ag-specific immunity when it was vaccinated with HA-Ag via the eye route.

### Comparison of the Poly(I:C)-Adjuvanted Inactivated Vaccine Efficacy between Eyedrop and IN Vaccination

**Fig 3:**
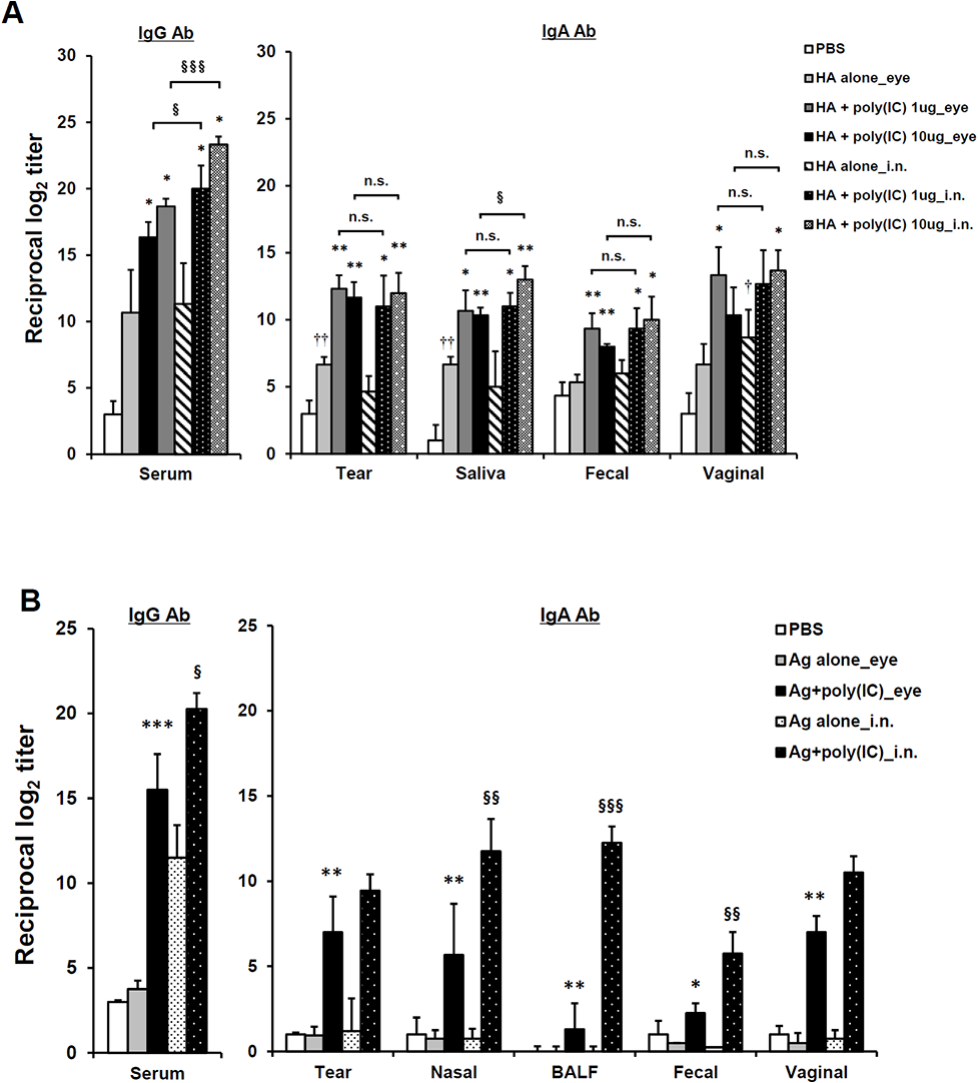
Comparison of Ab production between intranasal and eyedrop administration routes. (A) Dose-dependent HA vaccine antigens were administrated with 10 μg poly(I:C) resolved in 5 μl PBS by drops on both eyes two times at a 2-week interval in female BALB/c mice. HA-specific Ab levels were measured in serum and in various mucosal secretions 2 weeks after final vaccination by ELISA. (B) 1 μg H1N1 split vaccine antigen plus dose-dependent poly(I:C) resolved in 5 μl PBS were vaccinated by drops on both eyes two times at a 2-week interval in female BALB/c mice. Ag-specific Ab levels were measured in serum and in various mucosal secretions 2 weeks after final vaccination by ELISA. * *p* < 0.05, ** *p* < 0.01, *** *p* < 0.001 versus Ag alone; § *p* < 0.05, §§ *p* < 0.01, §§§ *p* < 0.001 versus poly(I:C) group. Results are representative of three independent experiments, with four mice in each group.

Reportedly, vaccination via the intranasal route is the most effective, among mucosal routes [24]. Thus, to examine whether the adjuvant efficacy of poly(I:C) in the eyedrop vaccination is more effective than that of poly(I:C) administration via other vaccination routes, we compared the efficacy of the immunization of HA vaccine plus poly(I:C) adjuvant via the eye route with that of intranasal vaccination in mice. In Fig 3A, when the serum IgG Ab levels of the eyedrop and intranasal vaccination groups, which were administered the same amounts of poly(I:C) and HA vaccine, were compared, all of the intranasal vaccination groups showed significantly higher serum IgG Ab responses than their eyedrop vaccination counterparts. However, in the mucosal wash fluids, there was no significant difference in the levels of mucosal IgA Ab-production between the eyedrop and IN vaccination groups, except for saliva fluids. Additionally, the enhancement of IgA Ab production levels in all of the mucosal fluids from mice administered 1 μg poly(I:C) plus HA eyedrop vaccine was significantly similar to that in intra-nasally immunized mice.

Next, to evaluate whether the adjuvanticity of poly(I:C) in eyedrop vaccination is maintained when the influenza virus HA-vaccine antigen is replaced with commercially used inactivated split A/California/04/09 H1N1 (H1N1) influenza virus vaccine antigen in eyedrops, BALB/c mice were immunized with 1 μg of split H1N1 influenza vaccine plus 10 μg poly(I:C) via eyedrops or intranasally twice at a 2-week intervals. Two weeks after the final immunization, as shown in Fig 3B, H1N1 vaccine-specific serum IgG Ab production levels in intranasally immunized mice with poly(I:C) plus vaccine antigen were significantly higher than those for eyedrop vaccinated mice. In mucosal fluids, except tear and vaginal wash samples, Ab production levels in the intranasally vaccinated groups were significantly higher than those in the eyedrop groups. However, the levels of Ag-specific Ab production in the poly(I:C) adjuvanted eyedrop vaccinated group were still significantly higher than those in the eyedrop Ag alone treated groups. Therefore, these results indicate that the efficacy of poly(I:C) in eyedrop vaccination is sufficient for use in commercial influenza vaccine antigens, with an efficacy similar with or slightly weaker than that for IN vaccination.

### Eyedrop Split H1N1 Influenza Vaccine Plus Poly(I:C) Vaccination Can Protect Mice from Lethal Influenza Virus Challenge

**Fig 4:**
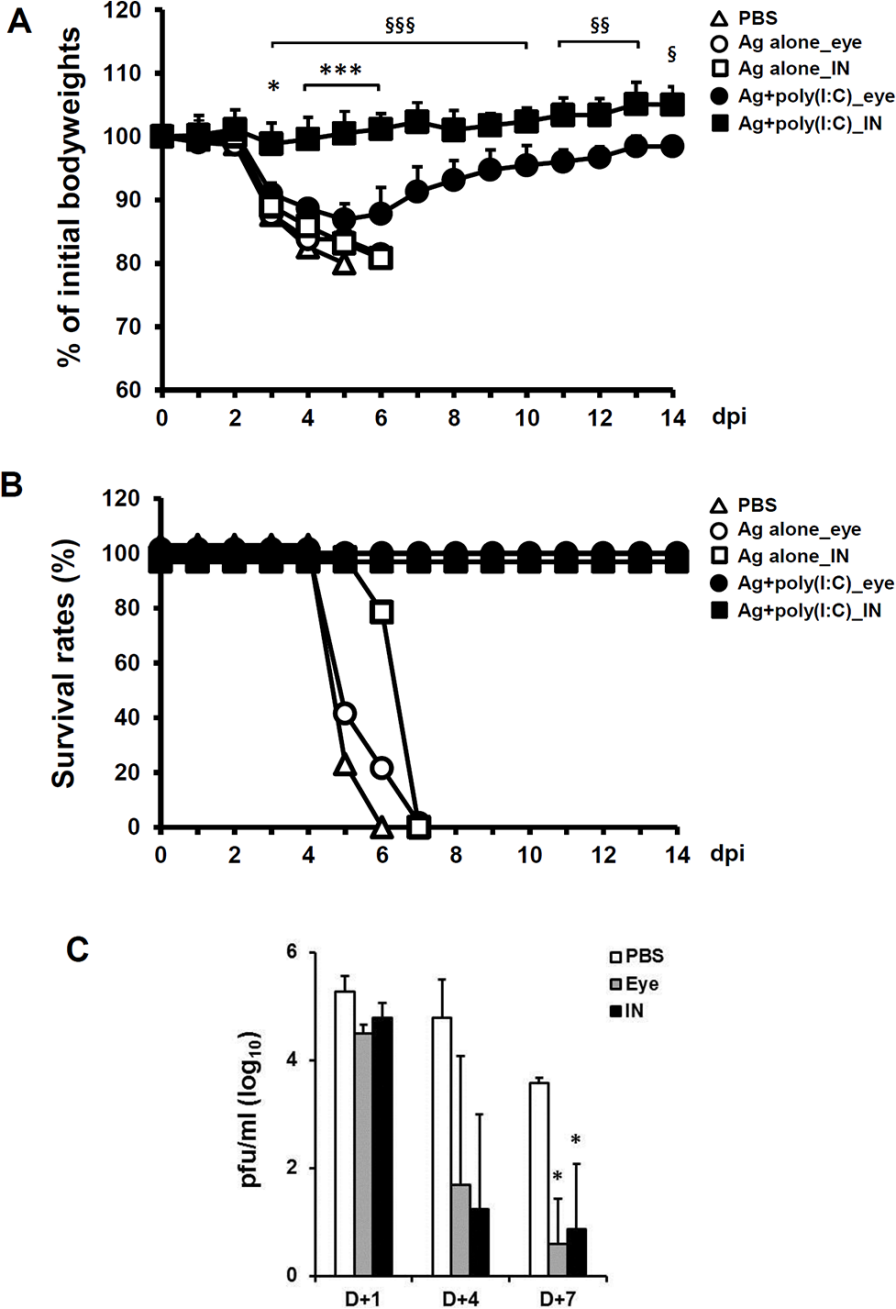
Eyedrop inactivated influenza vaccine plus poly(I:C) administration protects mice from lethal influenza virus challenge. Female BALB/c mice were given PBS (**△**) or H1N1 split vaccine Ag alone (eyedrop, ○; IN, □) or Ag plus 10 μg poly(I:C) (eyedrop, ●; IN, ■) by eyedrop or IN two times at a 2-week interval. Eyedrop groups were vaccinated with 5 μl of vaccine on both eyes and IN groups were received with 20 μl of vaccine. At 2 weeks after the last administration, mice were challenged IN with 10X LD_50_ of homologous mouse-adapted H1N1 influenza virus. Body weights (A) and survival rates (B) were monitored daily. (C) Nine mice in each group were vaccinated by eyedrop or intranasal immunization with 1 μg of H1N1 split vaccine plus 10 μg of poly(I:C). At two weeks after the final vaccination, mice were anesthetized and challenged with 50 μl of mouse-adapted live influenza A/California/04/09 (H1N1) virus suspension (10 X LD50; 0.75 TCID50) via the IN route. To measure the viral titers in the lung organs, three mice per group were sacrificed on day +1, +4 and +7. After the lungs were removed, they were homogenized in 1ml PBS using a small motor and upper respiratory tract was rinsed with 1ml PBS. Samples were centrifuged at 12,000×g and the supernatant fluids were removed, and then the supernatants were stored at −70°C until assayed for viral titers. Viral titers were assayed by plaque assay with MDCK cells. * *p* < 0.05; *** *p* < 0.001 between Ag plus 10 μg poly(I:C)_eye and PBS; § *p* < 0.05, §§ *p* < 0.01, §§§ *p* < 0.001 between Ag plus poly(I:C) treated eye and IN groups. Results are representative of three independent experiments, with five mice in each group.

To examine whether the immunity induced by vaccination of inactivated eyedrop vaccine plus poly(I:C) can protect mice from lethal influenza virus challenge, we vaccinated BALB/c mice with two doses of 1 μg of split H1N1 influenza vaccine plus 10 μg of poly(I:C) by eyedrops or IN as a positive control group at a 2-week intervals. Two weeks after the final vaccination, mice were challenged IN with mouse-adapted live influenza A/California/04/09 (H1N1) virus (10X LD_50_; 0.75 TCID_50_) and monitored for 2 weeks (Fig 4). Mice in the intranasally Ag + poly(I:C) treated group maintained their initial bodyweights throughout the monitoring period. Although eyedrop Ag + poly(I:C) treated mice lost about 10 percent of their initial bodyweight (Fig 4A), all mice began to recover their bodyweights on day 6 and survived against the lethal influenza virus challenge (Fig 4B). Meanwhile, PBS or Ag alone administered mice both by eyedrop and IN were critically affected by the influenza virus infection (Fig 4A) and all mice of PBS group and both Ag alone treated groups were sacrificed at humane endpoints (Fig 4B). Additionally, titers of the challenged influenza virus in lung tissue of mice were measured (Fig 4C). Proliferating viruses on day 1 began to reduce on day 4 in both the eyedrop and IN groups, and viruses were significantly cleared on day 7 in both vaccinated groups.

For an effective vaccine, the ability to induce long-lasting protection is an important requisite. To test whether the protective immunity induced by eyedrop vaccination with the split eyedrop H1N1 influenza vaccine is maintained for a long period of time, mice were vaccinated with the eyedrop H1N1 vaccine three times over a 1-week interval. At one year after the final vaccination, immunized Ag-specific Ab titers were examined with ELISA without additional boosting. The levels of Ag-specific IgG and IgA Abs in all samples, including serum and mucosal washes from mice immunized with eyedrop H1N1 Ag plus poly(I:C), were significantly higher than those of PBS or Ag alone treated groups (S1 Fig). Unfortunately, we cannot show survival data in which those vaccinated mice were challenged with homologous A/California/04/09 H1N1 influenza virus (10X LD_50_; 0.75 TCID_50_) since all mice in the PBS groups survived, even with the lethal influenza virus infection. Thus, these results suggest that the immunization of eyedrop inactivated influenza vaccine antigen plus poly(I:C) can elicit long-lasting Ag-specific humoral immunity, which was maintained for a year and the memory immunity might be enough to protect mice from influenza virus infection.

Additionally, we briefly compared the efficacy of the eyedrop vaccination with that over intramuscular (IM) vaccination (S2 Fig) since IM vaccination is most prevalent inoculation method. As shown in S2 Fig, Ag-specific serum IgG Ab levels in IM vaccinated groups were significantly higher than those of eyedrop group (S2A Fig), but nasal washes or BALF IgA Ag levels were not significantly increased than those of PBS. However, unexpectedly, after the lethal influenza H1N1 challenge there were almost no body weight loss in IM group (S2B Fig) and the differences in body weight changes between IM and poly(I:C) adjuvanted eyedrop group were significant (S2B Fig). Nevertheless, loss of body weights in eyedrop vaccine group was significantly lesser than those of Ag alone group and adjuvanted eyedrop vaccination protected mice from lethal influenza virus infection (S2C Fig). Through these results, although IM vaccine showed less body weight loss than eyedrop vaccine, eyedrop vaccine showed possibility that it can be alternatively used instead of IM vaccine with perfect protective efficacy of the protection of host from influenza virus infection.

### Safety of the Administration of Eyedrop Poly(I:C) Plus Inactivated Influenza Vaccines

**Fig 5:**
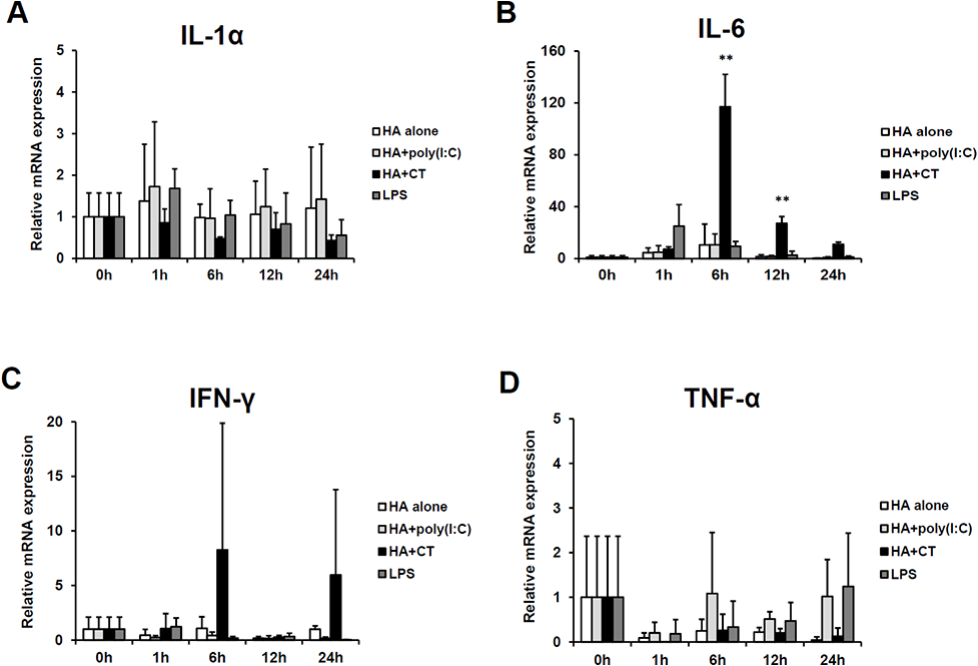
No inflammation in the eyes after administration of HA antigen plus poly(I:C) in mice. (A-D) Female BALB/c mice were administered 1 μg of HA vaccine alone, 1 μg HA plus 10 μg poly(I:C), or plus 2 μg CT resolved in 5 μl of PBS on the eyes for various time periods. Total RNA was extracted from homogenized conjunctival tissues with TRI reagents for reverse transcription and real-time PCR analysis. Gene expression levels were calculated as a relative ratio to the average value of house-keeping genes, β-actin. ** *p* < 0.01 versus 0 h. Data represent means ± S.D. of 3 independent experiments.

To examine the safety of inactivated influenza vaccine plus poly(I:C) in eyedrop vaccines, we checked whether the treatment with the eyedrop vaccine provoked inflammatory conditions in tissues of the conjunctiva in mice. For screening mRNA expression levels of inflammatory cytokines, BALB/c mice were given 1 μg HA vaccine antigen alone or plus 10 μg poly(I:C) or 2 μg CT for 1h, 6 h, 12 h, and 24 h (Fig 5A–5D). RNAs of the conjunctival tissues were extracted and mRNA of several inflammatory cytokines, such as IL-1 or IL-6, IFN-γ, and TNF-α, were quantified by qPCR. As shown in Fig 5, in all groups of mice, except the HA plus CT treated group, the mRNA expression levels of inflammatory cytokines at all designated time points were not up-regulated, compared to those at 0 h. Unexpectedly, LPS treatment did not induce any significant increase in mRNA expression levels of inflammatory cytokines (Fig 5B). Furthermore, we observed no histopathological changes in conjunctival and corneal tissues in LPS treated mice (data not shown). However, CT administration via eyedrops significantly induced about a 100-fold increase in IL-6 mRNA expression at 6h, which gradually decreased up to 24 h (Fig 5B). Also, IFN-γ mRNA was increased about 7-fold at 6 h after CT treatment, although the difference compared to mRNA levels at 0 h was not significant (Fig 5C). Otherwise there were no increases in inflammatory cytokine mRNA expression levels in corneal tissues nor those of IFN-α or IFN-β in corneal or conjunctival tissues at all designated time points (data not shown).

**Fig 6:**
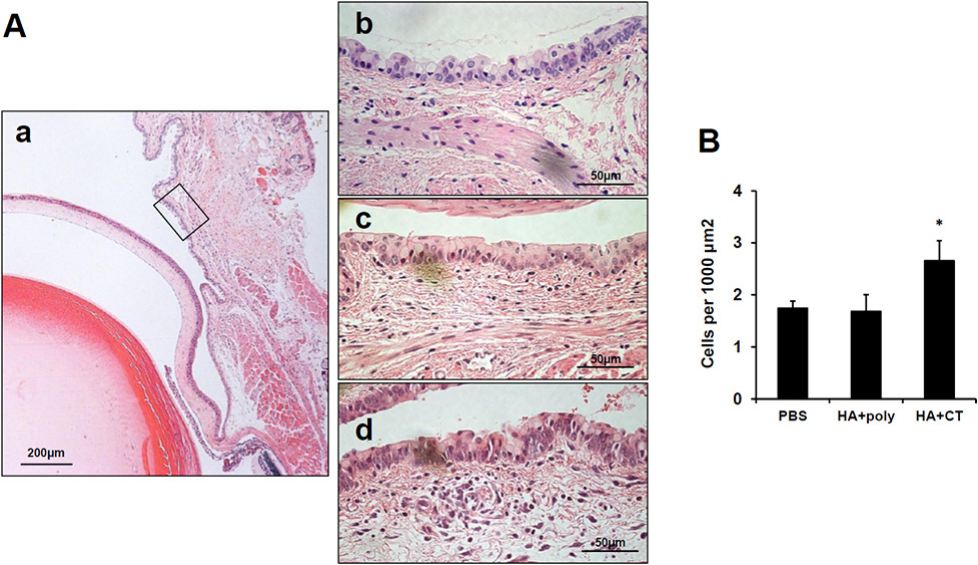
No induction of cellular infiltration in conjunctival tissues after administration of HA antigen plus poly(I:C). (A) Female BALB/c mice were administered PBS, 1 μg / 10 μl of HA vaccine antigens plus 10 μg /10 μl poly(I:C), or 2 μg/10μl CT on the eyes, and eye tissues were prepared and stained with H&E. Every representative figure of the same position of the rectangle (*Aa*) are shown (*Aa-b*, PBS; *Ac*, poly(I:C); *Ad*, CT). (B) Mononuclear cells were counted in sub-epithelial regions of tarsal conjunctival areas and plotted as a graph (cells per 1000 μm^2^). * *p* < 0.05; ** *p* < 0.01 versus Ag alone group. Results are representative of three independent experiments, with three mice in each group.

In histopathological assays of tissues of eyes after 24 h of 1 μg HA alone or HA plus 10 μg poly(I:C) or 2 μg CT eyedrop treatment, epithelial and goblet cells from the bulbar conjunctival to the tarsal conjunctival areas were intact and there was no symptom of hyperplasia in all conditions, including HA plus poly(I:C) or plus CT treatment, in both conjunctival (Fig 6Ab-d) and corneal tissues (data not shown). When mononuclear cells in sub-epithelial areas in all the conjunctival tissues were counted, excluding stromal cells, which have a distinctive long and narrow shape, there were no increases therein in the tissues of HA plus poly(I:C) treated eyes, compared to that of the PBS group (Fig 6B). However, as shown in Fig 6Ad, there were significantly more mononuclear cells in the tissues of HA plus CT administered eyes than those of PBS or poly(I:C) treated mice (Fig 6B), and the conjunctival tissues were slightly swollen after 24 h of CT treatment (data not shown). Since CT treatment on eyes provoked increased mRNA expression of IL-6 and IFN-γ (Fig 5B and 5C), we discerned that increased mononuclear cells had infiltrated to the areas upon increased expression or secretion of the inflammatory cytokines. Meanwhile, poly(I:C) treatment on the eyes for 24 h did not induce any cell infiltration in the conjunctival and corneal tissues.

**Fig 7:**
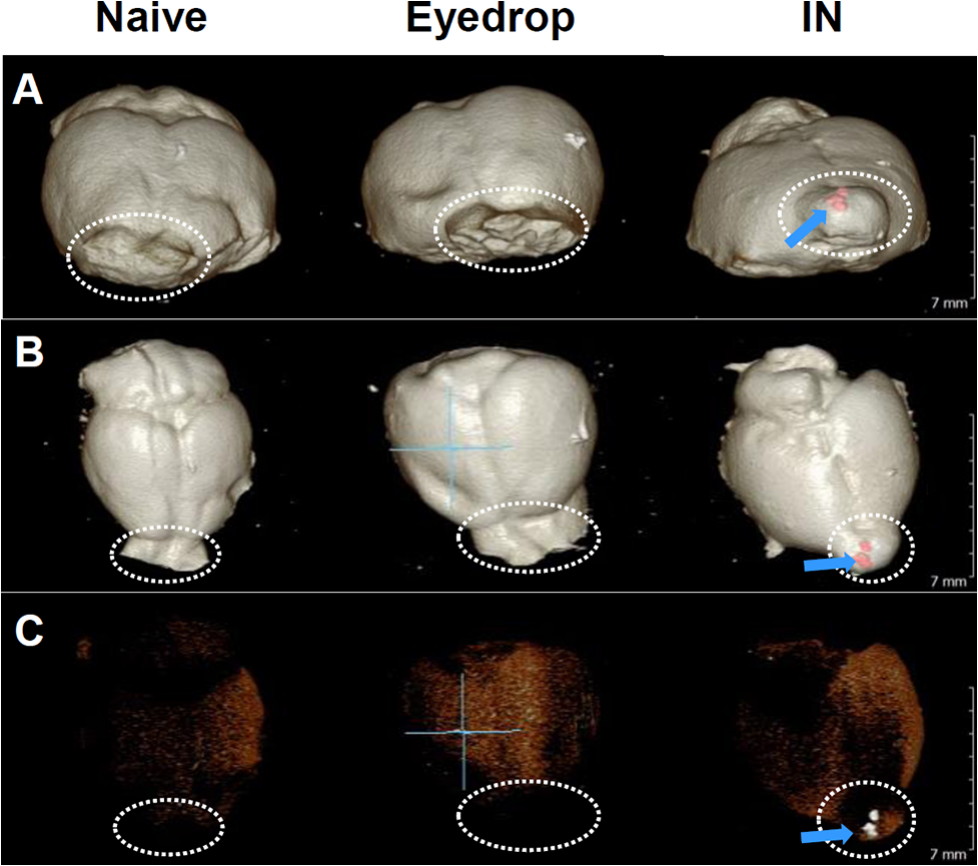
No detection of contrast medium on the olfactory bulbs from mice administered eyedrops. Female BALB/c mice were administered 10 μl or 50 μl of contrast medium by eyedrop or IN, respectively. After 30 min of contrast medium treatment, CT pictures of the brains of each mouse were taken. Frontal view (A), top view (B), or dimmed top view (C). Inner dashed circle, olfactory bulbs; blue arrows and red dots, spots of the contrast medium.

Additionally, since we previously showed that vaccinated eyedrop Ag did not redirect to the brain in the presence of CT, we attempted to visualize the presence of topically inoculated solution on the surface of the mouse brain with micro-computerized tomography (CT) scanning. The iodinated contrast medium was inoculated via eyedrops or IN in mice, and after 30 minutes, brains of each group were removed for micro-CT scanning. As shown in Fig 7, there was no spots of the contrast medium on the surface of brains from the naive or eyedrop groups. However, there were several spots (arrows) of the contrast medium on the OB region (dashed circles) of the brains from the IN treated mice. Thus, taken together, these results suggest that eyedrop inoculation is safe and effective, and may of use as an alternative to intranasal vaccination for inactivated influenza vaccine administration.

## Discussion

In this study, we showed that poly(I:C) is an effective eyedrop adjuvant of inactivated influenza vaccine in mice, eliciting enhanced immune response without adverse effects. Consistent with our previous study, in which the efficacy of eyedrop vaccine using live attenuated influenza virus was shown [6], inactivated influenza vaccine was enough to induce an enhanced immune response, and no local inflammation was observed in mice after eyedrop administration. Our results provide evidence that eyedrop vaccination of killed influenza vaccine is enough to safely induce protective immunity against influenza virus infection, and poly(I:C) can be used as a potent eyedrop vaccine adjuvant alternative to CT.

Herein, we evaluated the potential of killed virus antigens as an effective eyedrop vaccine in mice for the first time. Although the live attenuated influenza vaccines (LAIV) offers potentially greater immunity induction with smaller amounts of vaccine antigen than inactivated influenza vaccine by mucosa vaccination [3, 4], it can cause some adverse effects [3], and it allows the use of LAIV in restricted aged peoples [4, 25]. Thus, much research has been performed regarding the efficacy of killed influenza vaccine as an alternative to LAIV, along with studies on adjuvants that can be used to augment the activity of less efficient inactivated vaccines. Previously, we confirmed that eye mucosa is an alternative potent but safe mucosal vaccine administration route against influenza virus infection [6]. Thus, here, we attempted to evaluate the efficacy of inactivated vaccines adjuvanted by poly(I:C). HA subunit antigens and split H1N1 virus antigens were enough to induce strong immune response when poly(I:C) was used together (Fig 3). Since we could not prepare homologous HA subunits and split virus antigens derived from one virus strain, comparison of the immunity-inducing effect between those antigens was impossible. However, the potency of split vaccine induced-immunity was enough to protect mice from lethal (10 X LD_50_) homologous influenza virus challenge (Fig 4). Therefore, it can be suggested that eyedrop inactivated influenza vaccine can be used for influenza eyedrop vaccination as an alternative to LAIV, if it is adjuvanted with potent adjuvants, such as poly(I:C).

Along with the efficacy of killed virus antigens, poly(I:C) was examined as a potent adjuvant for eye mucosa. Although the efficacy of CT, the most effective mucosal adjuvant, is well reported, it causes severe adverse effect [7]. Thus, a study of alternative mucosal adjuvants other than CT, by which killed-influenza vaccine can be adjuvanted, is needed. Accordingly, we tested the efficacy of many conventionally used adjuvants for use in eye mucosa together with HA subunit antigen. When HA subunits and split influenza virus antigens were used with CT and poly(I:C), they induced significant enhancement of antigen-specific immune responses (Figs 1B and 2). Interestingly, the adjuvanticity of poly(I:C) was better when it was used with HA subunit antigens than OVA protein antigen. Poly(I:C) has once been shown as an effective adjuvant for use in nasal-mucosal vaccination against influenza virus [18]. Therefore, since eyedrop vaccination utilizes similar common cranial mucosal immune systems, poly(I:C) could also exert effective adjuvanticity along with inactivated influenza vaccine antigen in the eye mucosa (Fig 3B). Although alum and MPLA are potential adjuvants when they are used intramuscularly [26–28], their efficacy as an adjuvant in eye mucosa was as low as that of vaccine alone administration (Fig 1). Thus, we suggest that poly(I:C), which was as effective as CT for influenza vaccine inoculation, may be useful in eye mucosa vaccination as a potent adjuvant.

In the present study, eyedrop inactivated influenza vaccine was as effective as IN influenza vaccines. In many studies, it has been shown that intranasal or sublingual mucosa is an effective route for influenza vaccine immunization, and the efficacy of intranasally administered vaccine was shown to be similar to or better than that of sublingual influenza vaccine [29, 30]. However, intranasal administration of inactive influenza vaccine causes Bell’s palsy [8]. Thus, evaluation of other safe and effective alternative vaccination routes is required. Since IN is the most potent mucosal vaccination route for influenza vaccines, we evaluated the efficacy of killed vaccine in eye mucosa compared to that of intranasal vaccine. In the case of human papilloma virus vaccine, with as antigen amounts as low as 30 μg, the immunity inducing activity of intranasally vaccinated was more effective than those of other mucosal vaccination routes, (i.e., s.l., oral, i.m., i.vag., or rectal) [31]. In this study, we showed that the immunity-inducing effect of eyedrop influenza HA subunit vaccine was similar to that of the intranasal vaccine (Fig 3A). Although the efficacy of anti-H1N1 split vaccine antibody production tended to be better in IN vaccine than that of eyedrop, IgA Ab production levels in mucosal wash samples from both IN and eyedrop treated mice were similar (Fig 3B). Since protection against influenza virus challenge is exerted via secretion of IgA in mucosal barriers [32, 33], we suggest that vaccination via eyedrop inactivated influenza vaccines adjuvanted with poly(I:C) induces protective immunity against influenza virus infection, as much as IN vaccinations do.

IM vaccination is considered the most prevalently used vaccination method for the immunity induction against both systemic and mucosal viral infection. In our study, the efficacy of IM vaccine were confirmed again that even though mucosal IgA Ab levels in respiratory tract including nasal passage and lung airway of mice were lower than those of eyedrop vaccine treated group, IM vaccine showed less body weight loss than eyedrop vaccine and 100% protection from lethal influenza virus infection (S1 Fig). Although a host in which low Ag-specific IgA Ab levels in mucosal compartments were induced by IM vaccine seems to be vulnerable to mucosal virus infection, it has been shown that systemic IM vaccination successively induced Ag-specific cytotoxic cellular immunity in both systemic and mucosal compartments in both mice and rhesus monkeys [34, 35]. Nevertheless, it requires both needles to inject the vaccine and trained-health care provider to handle the needled syringes. Therefore, the requirements limit the range of the IM vaccine usage up to the area where medical workers can reach. In the context, eyedrop vaccine is suggested that it can be used as an alternative mucosal vaccine as it effectively protected hosts from lethal influenza virus challenge although impeding efficacy of body weight loss were better in IM vaccine group (S1B Fig).

Using low amounts of antigen in vaccines is an ideal goal of vaccine research, since it is correlated with less induction of unexpected side effects. Thus, several types of influenza vaccines have been evaluated in order to use fewer amounts of antigens. For intranasal HA subunit influenza vaccine, the effective minimum amount of antigen is 1 μg for one dose, when it is treated with 10 μg of poly(I:C) adjuvant [18]. In the eyedrop vaccine, likewise, the least effective amount of HA subunit or H1N1 split virus vaccine antigen was 1 μg, when it was immunized with 10 μg of poly(I:C) twice at a 2-week interval, to significantly enhance antigen-specific Abs production (Fig 2A). In regards to the use of poly(I:C), two doses of 1 μg poly(I:C) was enough to induce significantly enhanced antigen-specific Abs production together with 1 μg of HA subunit antigen. Moreover, eyedrop vaccination with 1 μg of split H1N1 influenza vaccine with 10 μg of poly(I:C) was enough to protect mice from lethal influenza virus challenge (Fig 4). Therefore, we suggest that eyedrop vaccination of influenza vaccines is as effective as IN vaccination. Moreover, vaccination via eye mucosa requires no greater amounts of antigen and adjuvant than those of IN route for inducing the same level of immunity.

With regard to safety, poly(I:C) could be suggested as a safe eyedrop adjuvant, with almost no possibility of evoking side effects on the eyes. Daily administration of poly(I:C) in nasal cavities, and even in the brains, of mice showed no adverse effects on the treated tissues, and mice maintained normal body weights and exhibited intact tissue histology [18]. Likewise, in this study, eyedrop influenza vaccine plus poly(I:C) administration induced neither inflammatory cytokine mRNA expression (Fig 5) nor infiltration of mononuclear cells (Fig 6) in conjunctivas after poly(I:C) treatment. Additionally, the safety of the use of polyICLC, an RNase-resistant analogue of poly(I:C) stabilized with poly-L-lysine, has been reported in several human clinical trials [20, 36, 37]. According to these studies, i.m. or s.c. administration of polyICLC was enough to elicit protective immunogenicity as much as poly(I:C) without adverse effects. Meanwhile, as shown in Figs 5 and 6, the presence of CT human monocytes reportedly differentiated into CD14^high^CD1^low^ macrophage-like DCs, induced increases in IL-1-, IL-6 and IL-10, as well as decreases in TNF-α and IL-12 [38]. Therefore, it seems that in eye mucosa, poly(I:C) involves a mechanism by which protective-immunity induction is initiated without inducing increases in inflammatory cytokine mRNA expression and cellular infiltration in conjunctival tissues. Meanwhile, the mechanism of CT is different from that of poly(I:C) in that its adjuvanticity is exerted by recruiting mononuclear cells accompanied with IL-6 cytokine production [38]. Thus, based on previous studies and our results, we suggest that polyICLC, a safe derivative of poly(I:C), may be of use as an alternative to poly(I:C). Also, the efficacy of poly(I:C) as a safe eyedrop vaccine adjuvant should be further evaluated in clinical trials of inactive eyedrop influenza vaccines.

Furthermore, as an alternative to intranasal vaccination, eye mucosa is a safe vaccination route. Firstly, the mechanism of immune privilege in the eyes guarantees the use of eyedrop vaccine. In the eyes, the immune privilege suppresses sight-damaging inflammation. Even if LPS is injected in vitreous cavities of the eyes in BALB/c mice to evoke intraocular inflammation, the intensity of inflammation declines after at 9 h, followed by immune privilege; even the growth of injected tumor cells is not suppressed by intraocular immunity [39]. Consistent with these results, we showed that eyedrop LPS treatment on the eyes does not evoke increases in inflammatory cytokine mRNA expression (Fig 5). Secondly, the eye route is an anatomically safe entry site for vaccine administration. The eye route comprises a tear drainage system, in which tears are constantly produced and drained away through tear ducts to protect the eyes from foreign particles that contaminate eye mucosa. Therefore, once vaccine solution is applied by eyedrops on the eyes, most of the solution drains into the nasal cavity via the punctum and nasolacrimal ducts with normal tear drainage, and then, finally, it is swallowed [15]. By virtue of the drainage system, deleterious effects provoked by topically applied materials on eye tissues, including the lens, retina, optic nerve, and so on, can be avoided.

Lastly, the vaccination of inactivated antigens by nasal administration has a potential to induce side effects by which nerve cells can be damaged. Previously, it was shown that OB epithelial cells can uptake proteins that their size are up to approtaximately 66 kDa and accumulates it in nerve cells [40, 41]. Additionally, our group showed that intranasally administered acridinium-labeled OVA was detected on OB [6] and the size of OVA is 45 kDa [42]. Since the size of inactivated influenza vaccine antigen molecules, such as HA or neuraminidase glycoproteins, or matrix protein, M1, ranges from 29 to 70 kDa, there is a possibility that intranasally administered inactivated influenza vaccine antigens or adjuvant molecules can be taken up by OB epithelial cells and it might exert damage effect on nerve cells which are connected with OB epithelial cells. In fact, it has been confirmed that eyedrop vaccine antigen is not redirected to the CNS in the presence of CT in mice. In contrast, intranasal administration of the acridinium-labeled OVA alone resulted in contamination of the OB in mice [6]. Thus, intranasal vaccination of inactivated antigens, as well as CT, has deleterious potential to induce unexpected side effects, especially on facial nerve cells.

In conclusion, this study was the first to show that the eye mucosa is a safe and potent immunity inductive site, even for the use of inactivated influenza vaccine when it is adjuvanted by poly(I:C). Since pathogen originated vaccine antigens show species-specific or tissue specific immune responses, strategies of eyedrop inactivated vaccine antigens of various pathogens could be established based upon this study.

## Conclusion

Although intranasal influenza vaccine is the most effective mucosal vaccine for influenza prevention, inactivated vaccines should be given only by muscular injection, and live-attenuated influenza vaccines (LAIV) are not allowed for use in vulnerable people. Recently, we reported that eyedrop LAIV is effective in intranasal vaccination. This study aimed to examine the possibility of using eyedrop inactivated influenza vaccine in mice as an effective and safe alternative to mucosal influenza vaccine. The eyedrop vaccine adjuvanted with poly(I:C) induced strong immunity, enough to protect mice from lethal influenza challenge, eliciting no inflammation, unlike CT adjuvant. Moreover, eyedrop vaccination resulted in no CNS contamination, whereas intranasal vaccination defiled the olfactory bulb of mouse brains. This study is the first to demonstrate the efficacy and safety of eyedrop inactivated influenza vaccine in mice, and we believe that this will provide new vaccine strategies for potent mucosal vaccination.

## Supporting Information

S1 Fig Comparison of the efficacy of immunity induction between IM and eyedrop vaccination. Female BALB/c mice were given PBS (□) or 1μg of H1N1 split vaccine Ag alone (eyedrop, ○) or 1μg Ag plus 10 μg poly(I:C) (eyedrop, ●) or 1μg Ag plus Imject (IM, ■) two times at a 2-week interval. At 2 weeks after the last immunization, Ag-specific Ab levels were measured in serum and in various mucosal secretions by ELISA, and mice were challenged IN with 10X LD_50_ of homologous mouse-adapted H1N1 influenza virus. Body weights (A) and survival rates (B) were monitored daily. * *p* < 0.05; ** *p* < 0.005 versus PBS (A). * *p* < 0.05; ** *p* < 0.005; *** *p* < 0.001 between Ag+Imject_IM and Ag+poly(IC)_eye or § *p* < 0.05 between Ag+poly(IC)_eye and Ag alone (B). Results are representative of two independent experiments, with five mice in each group.
(PDF)

S2 Fig Long-term Ag-specific Ab production induction in eyedrop vaccinated mice. Female BALB/c mice were given PBS, H1N1 split vaccine Ag alone, or Ag plus 10 ug poly(I:C) by eyedrop three times at a 2-week interval. At one year after the last immunization, Ag-specific Ab production levels were measured by ELISA (A). * *p* < 0.05; ** *p* < 0.01 versus PBS. Results are representative of two independent experiments, with five mice in each group.
(PDF)
