# Supplementary figures and images for: Inactivated Eyedrop Influenza Vaccine Adjuvanted with Poly(I:C) Is Safe and Effective for Inducing Protective Systemic and Mucosal Immunity
Source: PLoS One. 2015 Sep 10;10(9):e0137608. doi: 10.1371/journal.pone.0137608 (PMC4565664; doi:10.1371/journal.pone.0137608)

S1 Fig. Long-term Ag-specific Ab production induction in eyedrop vaccinated mice.

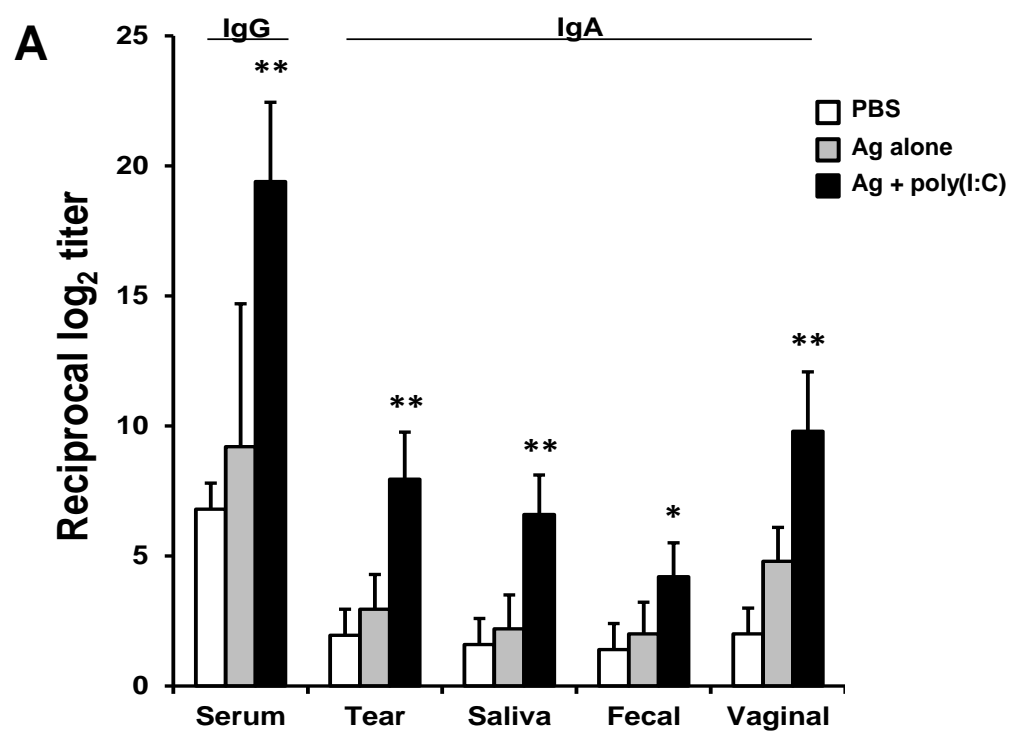

Supplement: S1 Fig — Female BALB/c mice were given PBS (□) or 1μg of H1N1 split vaccine Ag alone (eyedrop, ○) or 1μg Ag plus 10 μg poly(I:C) (eyedrop, ●) or 1μg Ag plus Imject (IM, ■) two times at a 2-week interval. At 2 weeks after the last immunization, Ag-specific Ab levels were measured in serum and in various mucosal secretions by ELISA, and mice were challenged IN with 10X LD50 of homologous mouse-adapted H1N1 influenza virus. Body weights (A) and survival rates (B) were monitored daily. * p < 0.05; ** p < 0.005 versus PBS (A). * p < 0.05; ** p < 0.005; *** p < 0.001 between Ag+Imject_IM and Ag+poly(IC)_eye or § p < 0.05 between Ag+poly(IC)_eye and Ag alone (B). Results are representative of two independent experiments, with five mice in each group. (PDF) [file pone.0137608.s001.pdf]

S2 Fig. Comparison of the efficacy of immunity induction between IM and eyedrop vaccination.

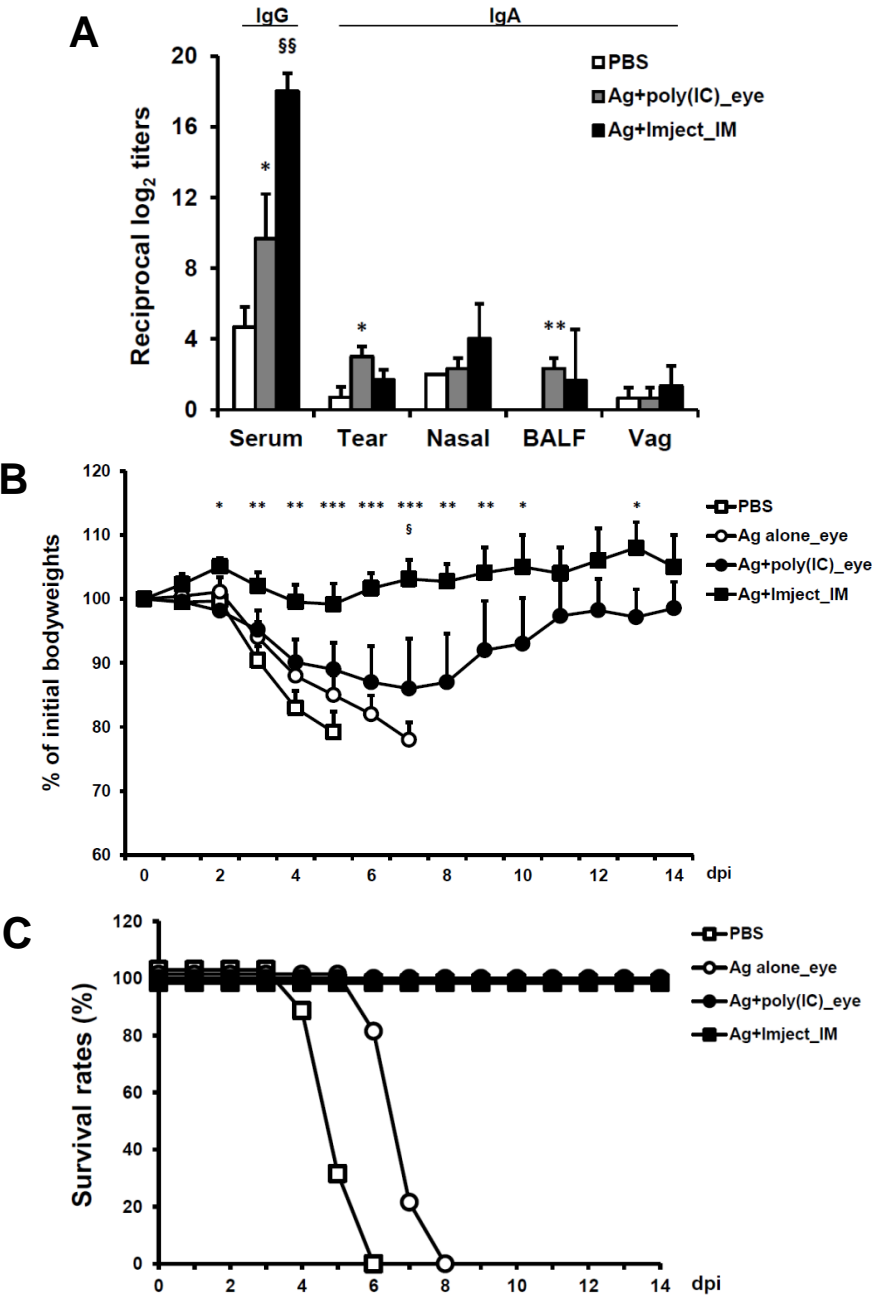

Supplement: S2 Fig — Female BALB/c mice were given PBS, H1N1 split vaccine Ag alone, or Ag plus 10 ug poly(I:C) by eyedrop three times at a 2-week interval. At one year after the last immunization, Ag-specific Ab production levels were measured by ELISA (A). * p < 0.05; ** p < 0.01 versus PBS. Results are representative of two independent experiments, with five mice in each group. (PDF) [file pone.0137608.s002.pdf]
